# Mental health, sleep quality, and hormonal circadian rhythms in pregnant women with threatened preterm labor: a prospective observational study

**DOI:** 10.1186/s12884-023-05801-4

**Published:** 2023-07-07

**Authors:** Xiao-Juan Wang, Xiao-Ting Li, Na Chen, Long Huang, Shui-Xiu Huang, Ting-Ting Chen, Gui-Hua Liu, Rong-Fang Hu

**Affiliations:** School of Nursing, Fujian Medical University, 1 Xue Yuan Road, University Town, Fujian Fuzhou, China

**Keywords:** Threatened preterm labor, Depression, Anxiety, Sleep, Actigraphy, Circadian rhythm, Melatonin, Cortisol

## Abstract

**Background:**

Threatened preterm labor (TPL) is an important obstetrical challenge. Pregnant women with TPL may develop psychological and physical problems such as mental health disorders, sleep disturbance, and hormonal circadian rhythm disruption. This study aimed to investigate the current state of mental health, sleep quality, and circadian rhythms of cortisol and melatonin secretion in pregnant women with TPL and normal pregnant women (NPW).

**Methods:**

A prospective observational clinical study was conducted at a maternal and child health hospital in Fuzhou, China, between June and July 2022. A total of 50 women between 32 and 36 weeks of gestation (TPL group, *n* = 20; NPW group, *n* = 30) were recruited. Data on anxiety symptom (Zung’s Self-rating Anxiety Scale, SAS), depression symptom (Edinburgh Postnatal Depression Scale, EPDS), subjective sleep quality (Pittsburgh Sleep Quality Index, PSQI) and objective sleep outcomes (measured by actigraphy) of the pregnant women were collected at the time of enrolment. Salivary samples were collected once every 6 h (i.e., at 06:00, 12:00, 18:00, and 00:00) during 2 consecutive days to measure the circadian rhythm of hormone (cortisol and melatonin).

**Results:**

There were no differences found in the total scores of SAS, EPDS scores, subjective sleep quality between the TPL and NPW groups (*P* > 0.05). In contrast, significant differences were found in sleep efficiency, total sleep time, wake time after sleep onset, and average awakening time between the groups (*P* < 0.05). The circadian rhythm of melatonin secretion was disrupted in the TPL group (*P* = 0.350); however, it was maintained in the NPW group (*P* = 0.044). The circadian rhythm of cortisol secretion was disrupted in both groups (*P* > 0.05).

**Conclusions:**

In the third trimester of pregnancy, women with TPL suffer from poorer sleep quality and disruption of circadian rhythm of melatonin secretion compared with NPW. Nevertheless, there were no differences found in mental health (i.e., anxiety and depression) and circadian rhythm of cortisol secretion. Large-scale studies should be conducted to evaluate these changes in women with TPL.

**Trial registration:**

The study was registered from Chinese Clinical Trial Registry (Number: ChiCTR2200060674) on 07/06/2022.

## Background

Threatened preterm labor (TPL) is an important obstetrical challenge [1] and a global health problem. This condition may seriously affect pregnant women and their fetuses or infants. Approximately 25–45% of women with TPL will experience preterm birth [2, 3]. TPL is associated with a 33% increased risk of perinatal mortality and severe perinatal morbidity [4], short- and long-term complications in newborns [5], and substantial economic burden [6].

TPL is a stressful event, which is likely to trigger a biopsychological stress response [7]. Both anxiety and depression symptoms may be triggered by TPL diagnosis from a psychological perspective [8]. Possible reasons of stress in women with TPL include medical interventions, an unknown prognosis [9], fear of losing the unborn baby, or preterm birth with the associated risk of permanent impairment of the infant, own health risks, and separation of the partner, antenatal hospitalization [7]. A meta-analysis (including 18 studies) indicated that the incidence of depression and anxiety among women who were hospitalized antepartum for obstetric complications was 34% and 29%, respectively [10]. A cross-sectional study [11] showed that 42% of hospitalized women with TPL had high levels of stress. Moreover, a study in China [12] reported that women hospitalized due to TPL commonly experienced emotional burden, and 54.7% of them developed early postpartum depressive disorders.

Psychological distress and a poor sleep status during pregnancy may increase the risk of adverse birth outcomes, including preterm birth [13, 14]. Sleep disturbances often occur with mood disorders, are a risk factor for onset, exacerbation, of mood disorders which were supported by previous studies [15]. The secretion of catecholamine and adrenocorticotropic hormone, as well as the eventual increase of serum cortisol after sleep disorders, result in symptoms of physical and psychological stress [16].

Melatonin is secreted from the pineal gland and plays an important role in the regulation of the circadian rhythm and related functions, such as sleep–wake cycle, immune function, and mood [17]. Cortisol, as an important stress hormone, is one of the major hypothalamic–pituitary–adrenal (HPA) axis biomarkers secreted by the adrenal cortex. Both are biological markers of the circadian rhythm that their secretion follows the day-night cycle, with melatonin secretion normally low during daytime, increasing at night, peaking in the middle of the night and decreasing in the early morning hour [18], while cortisol levels tend to run in an opposite pattern [19]. Changes in cortisol levels in the peripheral fluid are a result of the brain’s response to stress, and circulating cortisol concentrations vary depending on the mood of the individual [20]. A prospective cohort study of 157 pregnant women with TPL diagnosis showed that middle- and high-cortisol levels in women with TPL diagnosis before 29 weeks of gestation predicted earlier birth date [8]. Previous empirical studies revealed that both hormones showed variations in their rhythmical secretion according to the individual’s sleep state [21].

In China, TPL accounts for approximately 15% of high-risk pregnancies [22]. Following the universal two-child policy implemented in China since October 2015, the incidence of TPL among women with advanced maternal age increased [23]. To our knowledge, few studies have investigated the mental health, and no studies have yet evaluated the sleep–wake cycle, the concentration and circadian rhythm of cortisol and melatonin in pregnant women with TPL. It is necessary to comprehensively understand the mental health, sleep quality, and circadian rhythm of pregnant women for the protection of maternal health and infant development.

We hypothesized that TPL may result in biopsychological responses in pregnant women. Such responses may include disruption of the circadian rhythm of hormone secretion (i.e., salivary cortisol and melatonin), development of anxiety and depression symptoms, and disturbance of sleep (i.e., perceived sleep quality and actigraphy sleep outcomes). To test this hypothesis, a prospective observational study was conducted in pregnant women with TPL diagnosis and NPW between 32 and 36 weeks of gestation.

## Methods

### Study design and participants

A prospective observational clinical study was conducted at a maternal and child health hospital in Fuzhou, China, between June and July 2022. Pregnant women diagnosed with TPL (TPL group) and NPW (NPW group) were recruited from the maternity clinics and wards, respectively. The inclusion criteria were: (1) age > 18 years; (2) ≥ 32 and < 37 weeks pregnant; and (3) diagnosis of TPL upon admission at the hospital for women in the TPL group. Exclusion criteria were: (1) previous diagnosis of psychiatric disorders; (2) fetuses with deformity or defect detected by ultrasound; (3) previous severe obstetric complications (e.g., intrauterine growth restriction, placenta abruption, preeclampsia); and (4) sedatives or hypnotics were used during hospitalization. This study was approved by the Fujian Medical University Research Ethics Boards and registered at the Chinese Clinical Trial Registry (No. ChiCTR2200060674). All participants provided written informed consent prior to enrolment in the study. The participants could withdraw from the study at any time.

### Sample size

G-power Version 3.1.9.4 was used to calculate the sample size [24]. On the basis of our preliminary experiment results, the effect size of 1.15 was adopted for the sample size calculation. With a power of 0.90, an alpha of 0.05 (two-sided), the calculated sample size was 34 pregnant women (17 in each group). After adjusting for an attrition rate of 20%, the final required sample size was 40 (20 in each group).

### Salivary sample collection and measurement

For each participant, salivary samples (2 ml) were collected once every 6 h (i.e., at 06:00, 12:00, 18:00, and 00:00) during 2 consecutive days. At the time of enrolment, a researcher instructed the participants on the method of sample collection and storage. Saliva was collected using the sterile cotton ball-soaking method. The participants were requested to refrain from eating or brushing their teeth for 30 min prior to sample collection. For the collection, the participants were instructed to place one cotton ball under their tongue for 3–5 min until it was moist, and squeeze it into the marked black test tube while wearing sterile gloves. Thereafter, the samples were transferred to a refrigerator (− 80 °C) by the researcher for subsequent analysis of hormone concentration. Cortisol was measured using a cortisol competitive enzyme linked immunosorbent assay (ELISA) kit (MULTISCIENCES, Hangzhou, China), while melatonin was measured using a melatonin ELISA kit (IBL International GmbH, Hamburg, Germany).

### Circadian rhythm parameters

Saliva cortisol and melatonin levels were dynamically measured every 6 h (i.e., at 06:00, 12:00, 18:00, and 00:00) on eight time-points. Circadian rhythm parameters were calculated based on cosinor regression y = a + b × cos (x × π/12 − c × π/12), in which a, b, and c represent mesor, amplitude, and acrophase [25, 26], respectively. The mesor is the mean of all values across the circadian rhythm [26]. The amplitude is half the difference between the highest and the lowest points of the cosine function [26]. The acrophase represents the time point when the circadian cycle reaches the peak value [26].

### Instruments

#### Demographic data

A standard demographic questionnaire was used by uniformly trained researchers to collect demographic data of pregnant women, such as age, gravidity, parity (number), education, profession, body mass index, gestational week, type of medical insurance, household monthly income per person, address, and type of conception.

### Psychological assessment

Depression was assessed using the Chinese version of the Edinburgh Postnatal Depression Scale (EPDS) [27, 28] at the time of enrolment. The EPDS is the most commonly used self-report questionnaire to screen for perinatal depression among maternal women. This questionnaire consists of 10 items. Participants rated their feelings of depression using a four-point scale (0–3). The total score ranged 0–30, with higher scores indicating a higher likelihood of developing depression. A score of 10 was set as the cutoff value denoting symptoms of depression [27, 29]. The Chinese version of EPDS has demonstrated good reliability and validity (Cronbach’s α value: 0.79; half-coefficient: 0.76) [29].

Anxiety was assessed using the Chinese version of Zung’s Self-rating Anxiety Scale (SAS) [30, 31] at the time of enrolment. This is a 20-item self-report questionnaire that covers a variety of anxiety symptoms, both psychological and somatic present in the previous week. It utilizes a four-point Likert scale, with scores ranging from 1 (none, or a little of the time) to 4 (most, or all of the time). The numbers of reverse scoring items are 5, 9, 13, 17, and 19. The raw score of SAS is the cumulative score of each item. The standard score is the raw score multiplied by 1.25. The Chinese version of the SAS has demonstrated good reliability and validity (Cronbach’s α value: 0.80) [32].

### Sleep measurements

Subjective sleep quality was assessed using the Chinese version of the Pittsburgh Sleep Quality Index (PSQI) [33]. PSQI is a self-rating scale used to assess sleep quality over the past month. The scale contains seven subscales: subjective sleep quality (item 6), sleep latency (items 2 and 5a), sleep duration (item 4), habitual sleep efficiency (items 1, 3, and 4), sleep disturbance (items 5b − 5j), use of sleep medication (item 7), and daytime dysfunction (items 8 and 9). Each subscale is scored from 0 to 3. A global PSQI score is calculated by summing the scores of the seven subscales. A total score > 5 indicates poor sleep quality. The Chinese version of the PSQI has demonstrated good reliability and validity (Cronbach’s α value: 0.89) [33].

Sleep–wake patterns were determined using a watch-sized actigraphy device (WGT3X-BT; ActiGraph, LLC, USA) placed on the non-dominant wrist of the participants each day and night, except when bathing, for 2 consecutive days. Participants were instructed to wear the actigraphy device. The obtained data reflected the objective sleep quality, including sleep onset latency, sleep efficiency, total sleep time, wake time after sleep onset, the number of awakenings and average awakening time. Wake time after sleep onset refers to the minutes of a participant was awake between sleep onset and sleep offset; Number of awakenings refers to the count of instances when the participant woke up (for 1 or more minutes) during the sleep; Average awakening time is the average number of minutes the participant was awake per episode of awakening during the sleep. Moreover, participants were asked to record the time they went to bed and woke up each day. The data recorded by the actigraphy device were downloaded and analyzed using the ActiLife software (Version 6.1 1.4; ActiGraph, LLC, USA).

### Statistical analysis

Statistical analyses were performed using the SPSS software (version 20.0; SPSS Inc., Armonk, NY, USA). Missing items in the questionnaires were not included in the summed scores, and single questionnaires with > 20% missing items were discarded. Hormone measurements were performed using data from participants who completed all collections of saliva samples. Descriptive statistics were used to summarize baseline demographics and outcomes. Normal distribution was tested using the Kolmogorov–Smirnov test. Categorical variables were presented as frequencies with percentages. Continuous data with a normal distribution were presented as the mean ± standard deviation (SD). Continuous data with a non-normal distribution were presented as the median and quartile. The between-group differences were compared with a chi-squared test for categorical variables, an independent-samples* t*-test for continuous variables, or a Mann–Whitney *U* test for the non-normal variables. A 5% level of significance was used in the present study, and two-sided *P*-values denoted statistically significant differences. A 24-h period cosine curve fits were performed in Python (version 3.9, Python Software Foundation) using a Non-linear least squares method. The significance of the circadian fit was assessed by a Pearson correlation test with 95% confidence. For *P* < 0.05, circadian rhythmicity was considered significant.

## Results

### Participant characteristics

Of the 68 women with TPL admitted to the obstetric ward during the study period, 40 were eligible for inclusion in the study. Twenty cases were excluded due to incomplete sample collection; thus, the number of participants included in the final analysis was 20. Thirty pregnant women who were admitted to the hospital during the same period for normal labor examination were included as controls. There were no significant differences in socio-demographic characteristics between the TPL and the NPW groups (Table 1).

**Table 1:** Participant characteristics

**Variable**	**TPL (*n* = 20)**	**NPW (*n* = 30)**	*X*^*2*^/ ***t***	***P***
**Age (years)**			0.062	0.803
< 35	18(90.0%)	25(83.3%)		
≥ 35	2(10.0%)	5(16.7%)		
**Gravidity (number)**			0.000	1.000
once	8(40.0%)	12(40.0%)		
more than once	12(60.0%)	18(60.0%)		
**Parity**			0.231	0.765
Nulliparous (0)	12(60.0%)	20(66.7%)		
Multiparous (1–3)	8(40.0%)	10(33.3%)		
**Education**			6.150	0.059
≤ Junior high school	1(5.0%)	4(13.3%)		
Senior high school	8(40.0%)	3(10.0%)		
University/College	11(55.0%)	21(70.0%)		
≥ master's degree	0	2(6.7%)		
**Profession**			4.098	0.268
Housewife	8(40.0%)	5(16.7%)		
Official	3(15.0%)	10(33.3%)		
Unofficial	8(40.0%)	13(43.3%)		
Self-employed	1(5.0%)	2(6.7%)		
**Body mass index (kg/m**^**2**^**)**	24.67 ± 2.05	25.46 ± 1.95	1.381	0.174
**Gestational age (week)**	33.25 ± 1.59	33.80 ± 1.45	1.267	0.211
**Type of medical insurance**			3.803	0.071
Resident	10(50.0%)	7(23.3%)		
Staff	10(50.0%)	23(76.7%)		
**Household monthly income per person**			3.100	0.381
< ¥3000	0	2(6.7%)		
¥3001-¥5000	8(40.0%)	6(20.0%)		
¥5001-¥8000	8(40.0%)	13(43.3%)		
> ¥8000	4(20.0%)	9(30.0%)		
**Address**			0.062	0.803
Town	2(10.0%)	5(16.7%)		
City	18(90.0%)	25(83.3%)		
**Type of conception**			/	0.140
Natural	20(100.0%)	26(86.7%)		
Assisted	0	4(13.3%)		

The categorical variables are expressed as n (%). Normal data are given as mean ± SD

### Mental health

The results did not show significant differences in psychological outcomes between the TPL and NPW groups (*P* > 0.05). The mean SAS score in the TPL and NPW groups was 41.20 (SD = 4.538) and 42.07 (SD = 7.066), respectively (Table 2). Of the 50 pregnant women in our study, 14% reported anxiety symptoms and 28% reported depression symptoms. Only one woman in the TPL group had a SAS score > 50 (indicating at least symptoms of anxiety) compared with six in the NPW group. The median EPDS score was 6.50 and 6.00, respectively. A total of seven women in the TPL group (35%) had an EPDS score > 10 (indicating at least symptoms of depression) compared with seven (23.34%) in the NPW group. The mean PSQI score was 5.75 (SD = 1.943) and 6.83 (SD = 2.321), respectively (Table 2).

**Table 2:** Anxiety, depression, and self-reported sleep

Variable	TPL (*n* = 20)	NPW (*n* = 30)	*Z/t*	*P*
**SAS score**	41.20 ± 4.538	42.07 ± 7.066	0.528	0.600
**EPDS score**	6.50(3.25 ~ 9.00)	6.00(3.00 ~ 7.75)	–0.289	0.773
**PSQI score**	5.75 ± 1.943	6.83 ± 2.321	1.722	0.091
Sleep quality	1.00(1.00 ~ 1.00)	1.00(1.00 ~ 2.00)	–1.232	0.218
Sleep latency	1.00(0.25 ~ 2.00)	1.00(1.00 ~ 2.00)	–0.986	0.324
Sleep duration	0.00(0.00 ~ 1.00)	1.00(0.00 ~ 1.00)	–1.022	0.307
Habitual sleep efficiency	0.00(0.00 ~ 0.00)	0.00(0.00 ~ 1.00)	–0.807	0.419
Sleep disturbance	1.00(1.00 ~ 2.00)	1.50(1.00 ~ 2.00)	–0.688	0.491
Use of sleeping medication	0.00(0.00 ~ 0.00)	0.00(0.00 ~ 0.00)	–0.816	0.414
Daytime dysfunction	1.00(1.00 ~ 2.00)	2.00(1.00 ~ 2.00)	–1.643	0.100

Normal data are given as mean ± SD, whereas non-normal data are expressed as median (25th percentile, 75th percentile)

### Sleep quality

#### Self-reported sleep

Regarding self-reported sleep measures, Table 2 shows that the PSQI scores of the participants were not significantly different between the two groups (*P* > *0.05*). There were no differences found between the two groups in sleep quality (Z = –1.232, *P* = 0.218), sleep latency (Z = –0.986, *P* = 0.324), sleep duration (Z = –1.022, *P* = 0.307), habitual sleep efficiency (Z = –0.807, *P* = 0.419), sleep disturbance (Z = –0.688, *P* = 0.419), use of sleeping medication (Z = –0.816, *P* = 0.414), and daytime dysfunction (Z = –1.643, *P* = 0.100) (Table 2).

### Actigraphy

Table 3 presents the baseline sleep characteristics determined from the 2 days of actigraphy monitoring and demonstrates the differences in sleep-awake patterns between the groups. The results of the Mann–Whitney *U* test did not show significant differences in sleep onset latency and number of awakenings between the two groups (*P* > 0.05). In contrast, significant differences were found in sleep efficiency (Z = 3.467, *P* = 0.002), total sleep time (Z = 2.478, *P* = 0.020), wake time after sleep onset (Z = –3.994, *P* < 0.001), and average awakening time (Z = –2.895, *P* = 0.004).

**Table 3:** Actigraphy variables

Variable	TPL (*n* = 14)	NPW (*n* = 14)	*Z/t*	*P*
Sleep onset latency (min)	2.50(0.38 ~ 16.00)	5.75(0.00 ~ 24.50)	–0.047	0.963
Sleep efficiency (%)	60.36 ± 11.58	76.00 ± 12.28	3.467	0.002
Total sleep time (min)	310.93 ± 69.86	370.68 ± 57.09	2.478	0.020
Wake time after sleep onset (min)	196.29 ± 60.32	105.18 ± 60.39	–3.994	< 0.001
Number of awakenings	25.71 ± 5.83	22.21 ± 11.11	–1.044	0.306
Average awakening time (min)	7.59(6.36 ~ 9.34)	3.89(3.47 ~ 6.79)	–2.895	0.004

Normal data are given as mean ± SD, whereas non-normal data are expressed as median (25th percentile, 75th percentile)

### Melatonin concentration and circadian rhythm parameters

**Fig. 1:**
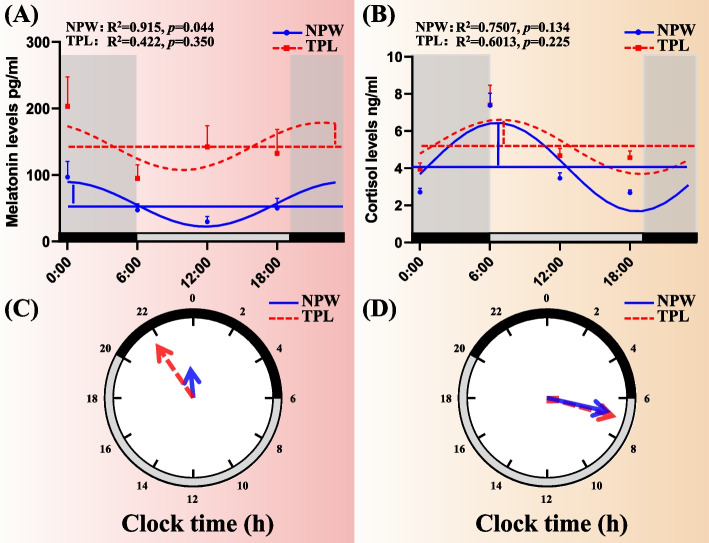
Circadian rhythm of saliva melatonin and cortisol secretion The upper part (**A**, **B**) was a cosine curve, and the lower part (**C**, **D**) was a continuous clock face from 00:00 to 24:00, as computed by the cosinor method. The last point in each clock was a duplicate of the start point, which was for visualizing periodically. And the data was subsequently smoothed with the cosinor method curve-fitting procedure (Python 3.9: Python Software Foundation). The acrophase was the phase of the maximal value assumed by the curve, and horizontal and vertical lines represented mesor and amplitude, respectively in figures (**A**, **B**). The goodness of rhythmicity (R^2^ and *p*–value) was shown on the top, and the black bar indicated the night or light-off period (20:00–06:00), and grey bars represented the day or light-on period (06:00–20:00) at the bottom of the lower figures (**A**, **B**). The amplitude and acrophase of a rhythm were plotted on a continuous clock face from 0:00 to 24:00, and the acrophase was indicated by the angle of a vector whose length corresponds to the amplitude in figures (**C**, **D**)

Circadian variations in melatonin secretion are illustrated in Fig. 1 and Table 4. The circadian rhythm of melatonin secretion was disrupted in the TPL group (*P* = 0.350); however, it was maintained in the NPW group (*P* = 0.044). Compared with the TPL group, melatonin levels, mesor values, and amplitude were significantly different in the NPW group (all *P* < 0.05). Repeated measures analysis of variance revealed a significant interaction between time (F = 4.522, *P* = 0.004) and group (F = 28.57, *P* < 0.001) in terms of melatonin levels (Table 5).

**Table 4:** Rhythm markers of cortisol and melatonin

Variable	TPL (*n* = 20)	NPW (*n* = 30)	*Z*	*P*
**Melatonin**				
Levels (pg/ml)	51.87(15.83 ~ 249.80)	23.62(13.77 ~ 39.64)	7821(U Value)	< 0.001
Mesor (pg/ml)	104.10(29.24 ~ 195.40)	31.46(18.41 ~ 68.25)	398(U Value)	0.001
Amplitude (pg/ml)	90.33(21.15 ~ 193.40)	18.43(7.96 ~ 68.37)	421(U Value)	0.003
Acrophase (h)	–0.02(–0.79 ~ 1.26)	0.17(–0.54 ~ 1.77)	608(U Value)	0.323
**Cortisol**				
Levels (ng/ml)	3.93(2.96 ~ 5.62)	2.78(2.25 ~ 4.02)	9454(U Value)	< 0.001
Mesor (ng/ml)	4.26(3.47 ~ 6.63)	3.71(2.59 ~ 5.16)	686(U Value)	0.027
Amplitude (ng/ml)	1.34(0.94 ~ 3.02)	1.82(0.55 ~ 4.33)	946(U Value)	0.962
Acrophase (h)	2.84(4.39 ~ 0.06)	4.34(2.67 ~ 4.59)	702(U Value)	0.037

Non-normal data are expressed as median (25th percentile,75th percentile)

**Table 5:** Melatonin and cortisol concentrations

Variable	Time	Group	Time*group
**Melatonin**			
Levels	F = 4.522, *P* = 0.004	F = 28.57, *P* < 0.001	F = 0.817, *P* = 0.485
**Cortisol**			
Levels	F = 30.400, *P* < 0.001	F = 10.200, *P* = 0.002	F = 1.280, *P* = 0.281

Time*Group: time means from the first to the last saliva collection and group means comparison between the two groups

### Cortisol concentration and circadian rhythm parameters

Circadian variations in cortisol secretion are presented in Fig. 1 and Table 4. The circadian rhythm of cortisol secretion was both disrupted in the TPL group (*P* = 0.225) and the NPW group (*P* = 0.134). Compared with the TPL group, cortisol levels, mesor values, and acrophase were significantly different in the NPW group (all *P* < 0.05). Repeated measures analysis of variance revealed a significant interaction between time (F = 30.400, *P* < 0.001) and group (F = 10.200, *P* = 0.002) in terms of cortisol levels (Table 5).

## Discussion

This prospective, observational clinical study compared the anxiety, depression, sleep quality, and hormone circadian rhythms between pregnant women with TPL and NPW. To our knowledge, this is the first study to evaluate and compare the concentration and circadian rhythm parameters of cortisol and melatonin in pregnant women with TPL.

### Mental health

Our results did not show significant differences in SAS and EPDS scores among pregnant women with TPL and NPW. Overall, 7 women (35%) in the TPL group had a score of 10 or greater on the EPDS in our study, and were thus identified as having antenatal depression. This rate is higher than that reported in Greece [34]. This finding scientifically responds to the on-going call for psychological intervention to prevent antenatal depression in pregnant women [35]. Generally, increased worry regarding life-threatening complications for the baby is associated with more severe symptoms of maternal anxiety or depression. Surprisingly, we found only one woman in the TPL group had a SAS score > 50 (indicating at least symptoms of anxiety) compared with six in the NPW group. Possible explanations for these observations are that hospitalized pregnant women felt more confident, worried less about their child’s health, and experienced less anxiety as their health problems were resolved. This is consistent with the results of another study [36]. In addition, we also consider the results may be affected by confounding factors, such as environmental change or humanistic care in the hospital. Pregnant women in our study experienced individual episodes of anxiety and depression. Of the 50 pregnant women, 14% reported anxiety symptoms; this rate is lower than that observed in the general population (28.8%). In addition, 28% of pregnant women reported depression symptoms; this rate is higher than that noted in the general population (16.5%) [37]. These findings suggested that attention should be paid to the mental health of pregnant women in the third trimester, particularly symptoms of depression.

### Sleep quality

Our results did not show significant differences in PSQI scores among pregnant women with TPL and NPW, indicating no difference in subjective sleep quality. Nevertheless, significant differences were found in sleep efficiency, total sleep time, wake time after sleep onset, and average awakening time based on the actigraphy data, indicating that NPW had better objective sleep quality than those with TPL. In our study, the mean total PSQI scores in both groups were > 5, suggesting that all women in the third trimester experience poor subjective sleep quality regardless of the presence of TPL. Similar to the study conducted by Zhou et al. [38]. All these suggested that the clinical staff should pay attention to the pregnant women in sleep quality, especially in the third trimester. The minutes of awaking between sleep onset and sleep offset, and the average number of minutes awaking per episode of awakening during the sleep, were higher in the TPL group versus the NPW group. The sleep efficiency was opposite. The findings of actigraphy data revealed that pregnant women with TPL had worse sleep quality than NPW, which supported our hypothesis. A possible explanation for this finding is that worry in pregnant women with TPL regarding the health of their children and environmental changes led to poor sleep quality.

In this study, there were differences in the subjective and objective results of sleep quality between the two groups, indicating low consistency between the two assessment methods. Low agreement between the PSQI and actigraphy measures was previously observed in a pregnant women sample [39]. The low agreement is likely related to the fact that actigraphy measures sleep in real time over several nights, whereas the PSQI asks women to retrospectively rate their sleep during the last month. Retrospective recall may hinder accuracy, as it could be impacted by several bad nights and other biases. Actigraphy should be used for 3 days or more to get more accurate results. However, in order to be consistent with the timing of hormone collection, only two days of the actigraphy were used in this study. We suggest that the objective sleep of pregnant women can be monitored for a longer time with the actigraphy in the actual clinical work in the future, so as to provide more accurate sleep quality.

### The circadian rhythm of melatonin and cortisol

The circadian rhythm plays an important role as the clock of the human body, regulating physiological changes according to a 24-h light–dark cycle [40]. In previous studies, melatonin and cortisol have been widely used to examine the circadian rhythm [21]. In view of the importance of circadian rhythm, the present study pay attention to the circadian rhythm of hormone secretion in pregnant women, but the limitation was that it did not focus on the hormone concentrations at a single time point. Our results of hormone analysis showed that the circadian rhythm of saliva cortisol secretion was disrupted in the third trimester of pregnancy. Although the circadian rhythm of melatonin secretion was disrupted in pregnant women with TPL, it was maintained in NPW.

Melatonin is thought to regulate the sleep/wake cycle in humans [41], and maternal melatonin is involved in fetal development [42]. In our study, the circadian rhythm of melatonin secretion was disrupted in the TPL group; however, it was maintained in the NPW group. A possible explanation for this observation is that the circadian rhythm of melatonin secretion in women hospitalized for TPL was disrupted by changes in nighttime light exposure (e.g., nighttime ward rounds and changes in sleep conditions). A systematic review revealed that light exposure affects the secretion of melatonin [43]. Moreover, a previous study demonstrated that women who developed comorbidities during pregnancy had lower daytime melatonin levels [18]. Our results differed; the levels of melatonin were higher in the TPL group versus the NPW group, possibly because we collected saliva from pregnant women throughout the day rather than only during daytime. Melatonin levels were low during the day, increased at night, peaking in the middle of the night, and gradually decreased thereafter [44]. Based on our results, the peak of melatonin secretion was observed at 22:00 in the TPL group and at 00:00 in the NPW group. The peak point of melatonin secretion was earlier in the TPL group versus the NPW group, which may be explained by the earlier sleep time of pregnant women in the hospital compared with that of NPW at home. The lack of activity and the suspension of work and study may have contributed to the earlier sleep time of pregnant women in hospital versus those at home. Thus, the difference in the concentration and secretion of melatonin between the TPL and NPW groups may be attributed to the stress induced by TPL, which affected the sleep habits of pregnant women. This study yielded preliminary findings with regard to the disruption of the circadian rhythms of cortisol and melatonin secretion in women diagnosed with TPL.

Cortisol is thought to be a valuable potential marker of stress [45], and maternal peripheral cortisol levels may affect fetal brain development [46]. Our results revealed that the circadian rhythm of cortisol secretion was disrupted regardless of the occurrence of TPL. A possible explanation for this rhythm disruption is that cortisol secretion is increased in pregnant women in the third trimester of pregnancy. As reported by Lazarides et al. [47], the levels of maternal cortisol increased with advancing gestation. A study suggested that women with negative expectancies concerning future stressful events may become more sensitive to such events, reflecting an exacerbated hypothalamic–pituitary–adrenal axis response to stress [48]. Similarly, pregnant women with TPL may have a negative view of their situation, which increases cortisol secretion. In the present study, the levels of cortisol were higher in the TPL group versus the NPW group. In addition, a previous study [49] found that poor subjective sleep quality in pregnant women was associated with higher concentrations of cortisol. This was consistent with our findings; the TPL group, which was characterized by poor sleep quality, showed higher cortisol concentration. Cortisol levels are highest between 7 a.m. and 8 a.m. [45]. This was consistent with our findings (i.e., the concentration of cortisol varied at different time points, and the peak of cortisol secretion in both groups was recorded around 07:00). It is suggested that the stressful event of TPL affected the mood and sleep of pregnant women, subsequently leading to the change in cortisol secretion. Nonetheless, further investigation is warranted to verify our results and identify the reasons for the disruption of the circadian rhythm of cortisol secretion in pregnant women with TPL.

### Limitations

Our study had several limitations. Firstly, the TPL group collected data in hospital, while the NPW group completed the collection at home, which may result in interference of outcome measurements by environment factors. Secondly, our investigation was a preliminary exploratory study with a small sample size and a single center. Additional studies with larger sample sizes are warranted to confirm the current findings.

## Conclusions

The present study did not reveal differences in anxiety, depression, and subjective sleep quality between pregnant women with TPL and NPW. However, objective sleep quality and the circadian rhythm of melatonin secretion differed between the two groups. The effects may be influenced by other factors, such as changes in the hospital environment and individual differences. Clinical staff should pay close attention to the sleep conditions and the disruption of the circadian rhythm of melatonin secretion in pregnant women with TPL. Certain measures (e.g., centralized related therapeutic procedures during the day, and reduction of procedures and exposure to light at night) may help improve the sleep quality and regulate the circadian rhythm of melatonin secretion in pregnant women diagnosed with TPL.

## Data Availability

Datasets used and analyzed during this study are available from the corresponding author on reasonable request.

## Abbreviation

TPL: Threatened preterm labor
NPW: Normal pregnant women
SAS: Self-rating Anxiety Scale
EPDS: Edinburgh Postnatal Depression Scale
PSQI: Pittsburgh Sleep Quality Index
